# Minimising carbon and financial costs of steam sterilisation and packaging of reusable surgical instruments

**DOI:** 10.1093/bjs/znab406

**Published:** 2021-11-28

**Authors:** Chantelle Rizan, Rob Lillywhite, Malcolm Reed, Mahmood F Bhutta

**Affiliations:** Ear, Nose and Throat Department, University Hospitals Sussex NHS Foundation Trust, Brighton, UK; BSMS Teaching Building, Brighton and Sussex Medical School, Brighton, UK; Research Department, Royal College of Surgeons of England, London, UK; Department of Life Sciences, University of Warwick, Coventry, UK; BSMS Teaching Building, Brighton and Sussex Medical School, Brighton, UK; Ear, Nose and Throat Department, University Hospitals Sussex NHS Foundation Trust, Brighton, UK; BSMS Teaching Building, Brighton and Sussex Medical School, Brighton, UK

## Abstract

**Background:**

The aim of this study was to estimate the carbon footprint and financial cost of decontaminating (steam sterilization) and packaging reusable surgical instruments, indicating how that burden might be reduced, enabling surgeons to drive action towards net-zero-carbon surgery.

**Methods:**

Carbon footprints were estimated using activity data and prospective machine-loading audit data at a typical UK in-hospital sterilization unit, with instruments wrapped individually in flexible pouches, or prepared as sets housed in single-use tray wraps or reusable rigid containers. Modelling was used to determine the impact of alternative machine loading, opening instruments during the operation, streamlining sets, use of alternative energy sources for decontamination, and alternative waste streams.

**Results:**

The carbon footprint of decontaminating and packaging instruments was lowest when instruments were part of sets (66–77 g CO_2_e per instrument), with a two- to three-fold increase when instruments were wrapped individually (189 g CO_2_e per instrument). Where 10 or fewer instruments were required for the operation, obtaining individually wrapped items was preferable to opening another set. The carbon footprint was determined significantly by machine loading and the number of instruments per machine slot. Carbon and financial costs increased with streamlining sets. High-temperature incineration of waste increased the carbon footprint of single-use packaging by 33–55 per cent, whereas recycling reduced this by 6–10 per cent. The absolute carbon footprint was dependent on the energy source used, but this did not alter the optimal processes to minimize that footprint.

**Conclusion:**

Carbon and financial savings can be made by preparing instruments as part of sets, integrating individually wrapped instruments into sets rather than streamlining them, efficient machine loading, and using low-carbon energy sources alongside recycling.

## Teaser

Carbon and financial savings can be made by opting to prepare instruments as part of sets rather than individually wrapped items in single-use polypropylene pouches, efficient loading of decontamination machines, and recycling. Streamlining instrument sets (by removing infrequently used instruments) counterintuitively increased the carbon footprint and financial cost. The absolute carbon footprint was dependent on the energy source used for decontamination, but this did not alter the optimal processes to minimize that footprint.

## Introduction

Around 313 million surgical procedures are performed annually worldwide^1^, with the global surgical equipment market growing by 7.8 per cent per year and anticipated to be worth €16.8 billion by 2025^2^. As a resource-intensive area, surgery has a considerable environmental impact, which can be evaluated using a carbon footprint to estimate the greenhouse gases (GHGs) associated with surgical products and supporting processes. Surgical equipment is typically a major surgical carbon hotspot in an operation^3^, and using a greater proportion of reusable rather than single-use equipment has been identified as a critical strategy to reduce overall environmental harm^4^. However, even for low-carbon procedures, such as a cataract operation in India using (predominantly) reusable products, the overall carbon contribution from equipment remains high (72 per cent; 57 per cent reusable products, 15 per cent single-use items)^5^. Understanding how to optimize the decontamination and packaging of reusable surgical equipment will help guide surgical teams in meeting carbon reduction targets in healthcare^6^.

Reusable surgical instruments are typically grouped into sets containing instruments required for a specified procedure or group of procedures, and placed in trays or baskets. After use, instruments are packaged and go through a process of decontamination, which involves cleaning and subsequent microbial inactivation by means of disinfection and/or sterilization^7^. Microbial inactivation is most often achieved using steam, although alternative low-temperature methods include use of ethylene oxide, vaporized hydrogen peroxide gas plasma, or ozone^7^. Packaging of instruments for decontamination employs sterile barrier systems, which permit permeation of the sterilization agent, but prevent post-treatment entry of microorganisms, to maintain sterility until the point of use^8^. Options include tray wraps (usually 1–3 single-use layers, typically made from polypropylene and paper), reusable rigid containers (usually made from aluminium, stainless steel or plastics, with or without a filter) or flexible pouches (thermally sealed sleeves made from combinations of paper and plastics).

An additional factor determining the carbon footprint of decontamination and packaging is the composition of instrument sets. In some surgical procedures, additional individually wrapped instruments (not included in the set) may be opened, which are subsequently repackaged and decontaminated. Conversely, some instruments in a set may remain unused in a particular procedure. There has been a recent drive to streamline instruments included in reusable sets^9–12^, whereby less commonly used instruments are removed from sets, and reclassified as individually wrapped instruments, to be opened only if specifically needed. Previous studies have suggested the potential for elimination of up to 60 per cent of instruments in a paediatric set^9^, and 39 per cent in minor urology sets by removal of instruments used less than 20 per cent of the time^10^. Consolidating instrument sets may reduce costs^9^^,^^10^, and the time spent assembling sets and undertaking perioperative checks^11^^,^^12^, but no studies have reported the impact on the carbon footprint.

In summary, the carbon footprint and financial cost of preparing surgical instruments for reuse is determined by the decontamination process, the sterile barrier system and the composition of instrument sets, and the need to open individually wrapped instruments.

Previous studies have evaluated the carbon footprint of steam sterilization of specific instruments: a central venous catheter (Australia)^13^, reusable ureteroscope (Australia)^14^, and reusable laryngoscope blade (USA)^15^. Electricity requirements have also been determined for decontamination of a caesarean section instrument tray (USA)^16^, and electricity and water requirements for decontaminating 1kg of linens and instruments (Australia)^17^.

To date, no studies have reported the carbon footprint and financial cost of different processes for decontamination and preparation of reusable surgical instruments, nor how such processes can be optimized. The aim of this study was to estimate and compare carbon and financial costs associated with different modelled scenarios for decontamination and packaging of surgical instruments, including streamlining instrument sets, in a regional UK hospital.

## Methods

### Study setting

Carbon footprint and financial costs were estimated from processes for decontamination and packaging reusable surgical instruments at the Royal Sussex County Hospital (RSCH), a publicly funded (National Health Service) regional hospital providing both elective and emergency operations across a range of surgical specialties. At RSCH, the Sterilization Services Department (SSD) provides services for the local network of public hospitals (alongside a smaller SSD at another site). In 2018–2019, around 62 000 procedures or interventions were performed annually across these hospitals. Around two-thirds of the items sterilized (April 2017 to March 2018) were instrument sets, and one-third individually wrapped items, with 85 per cent of the former housed in reusable containers and the other 15 per cent in single-use tray wraps.

### Calculation of carbon footprint

Carbon footprints were estimated in accordance with the *Greenhouse Gas*  *Accounting Sector Guidance for Pharmaceutical Products and Medical Devices*^18^. Process-based (bottom-up) carbon footprinting was used for all components, aside from the detergent, which was modelled using an environmentally extended input–output analysis (top-down). Emission factors were sourced from the UK Government GHG Conversion Factors for Company Reporting database^19^, Inventory of Carbon and Energy database (version 3)^20^, Small World Consulting Carbon Factors Dataset^21^, and the authors’ own study of healthcare waste^22^ (*Table S1*). The resulting carbon footprint was expressed as carbon dioxide equivalents (CO_2_e), which summates direct GHG emissions (for example, from burning fossil fuels at study site) and indirect emissions (such as electricity, purchased goods, and waste).

Units of assessment were one cycle of each decontamination machine, one surgical instrument (either as part of a set or individually wrapped), and one typically sized set (housed in a rigid container or tray wrap). Processes included were energy and materials required by the washer/disinfector, sterilizer, and sterile barrier system, and disposal of materials (*Fig. S1*). Capital goods, hospital infrastructure, and production and disposal of surgical instruments were excluded. Vehicular transportation was not required between operating theatres and the on-site SSD.

### Analysis for decontamination

At RSCH, there were two types of machine for decontamination: two washer/disinfectors (TW300/3; Steelco, Treviso, Italy) and four steam sterilizers (V9934; BMM Weston, Faversham, UK). The washer/disinfector machines had 12 slots (each with capacity to hold a standard-sized instrument set, with larger sets split across two slots), with instruments passing through three chambers each with a capacity of 400 litres, and reaching 90–95°C for around 1 min during the decontamination cycle. The steam sterilizer had 18 slots (each slot housing a standard to large set, or up to 2 small sets), with a capacity of 1250 litres, reaching 134–137°C for approximately 3 min. The number of individually wrapped instruments that could be housed in each slot depended on the size of those instruments.

Decontamination processes were determined through discussion with engineers, data managers, and senior staff at the SSD. Typical input requirements were determined for each machine per unit time of operation from technical specification sheets and direct contact with manufacturers; no primary data on energy or water consumption were collected. The mean duration of the machine cycle was determined across three cycles of each process and used to calculate process activity data per cycle. Typical machine-loading patterns were determined using a prospective audit of 10 cycles on one day to record the mean number of slots used, number of instruments per slot, and whether slots were filled with sets or individually wrapped items. The mean number of instruments per set was determined by retrospective audit of RSCH instrument decontamination over 1 year (1 July 2018 to 30 June 2019). The typical carbon footprint and cost per set were estimated using mean loading patterns and the mean number of instruments per set.

The cost of decontaminating instruments was based on the charge by the SSD to surgical departments per set and per individually wrapped instrument, measured in British pounds (£). Values were converted to euros (exchange rate 16 June 2021).

Scenario modelling and multiple linear regression was used to evaluate and compare carbon footprints and the cost of alternative processes for instrument decontamination, including different machine loading capacities (25, 50, 75, 100 per cent), and number of instruments per slot (5–30, at intervals of 5). The latter equates to the number of items in a standard set for instruments, or the number of items per decontamination machine slot for individually wrapped instruments. The carbon footprint of the lowest machine-loading pattern observed during the audit was also estimated.

### Sensitivity analysis of different sources of energy for decontamination

The SSD at RSCH used a natural gas-fuelled steam generator to supply steam for both the washer/disinfector and sterilization machine. The alternative of steam generated by electricity was modelled, as well as different sources of electricity and natural gas supply. Region-specific emission factors for electricity and natural gas were extracted from SimaPro Version 9.10 (PRé Sustainability, Amersfort, the Netherlands), using the Ecoinvent database (version 3.6).

For electricity, data were employed from: Australia, which uses a large proportion of non-renewable high-carbon energy sources (coal); Iceland, which uses predominantly low-carbon renewable energy sources (geothermal and hydropower); and regions that use a mix of sources (USA, European average, and global average). For natural gas, the carbon footprint of on-site combustion is relatively constant, but well-to-tank emissions show large geographical variations depending on upstream processes for extraction, refining, transportation, and transmission via pipelines^23^. Well-to-tank emissions were modelled for US, European average (also used for Icelandic models owing to lack of country-specific data), and global average calculations (also used for Australian models). The water supply and detergent were responsible for less than one per cent of the decontamination carbon footprint and so were not altered in this analysis.

### Analysis of sterile barrier systems

Three alternative packaging materials recommended by WHO guidance^24^ were compared with modelling based on specific examples available at the RSCH. Set instruments were placed in a reusable stainless-steel mesh basket, and in the first scenario housed in reusable aluminium containers, and in the second scenario within two layers of single-use tray wrap made from polypropylene and paper. In the third scenario, instruments were packaged individually in two single-use flexible peel pouches, each made from paper on one side and polyethylene on the other. Associated packaging and labelling of instruments were included for all scenarios.

The carbon footprint of each scenario was estimated by determining the material composition of each sterile barrier system (from manufacturer information where available, or expert knowledge), and weighing each component (FPRS4202 precision balance scales; Fisher Scientific, Loughborough, UK). The lifetime of stainless-steel baskets was estimated at 10 years, and plastic identification tags at four years (expert opinion of SSD senior staff and industry contacts), with a mean of 11.6 uses per year for both components (based on a one-year retrospective audit of RSCH decontamination of 67 080 instrument sets). Reusable aluminium containers were assumed to be used 1000 times, in accordance with manufacturer information. Reusable containers were washed in dedicated cycles, with each washer/disinfector machine loaded with six containers per cycle, and the impact of this included and assigned to the packaging. However, instruments were sterilized within their packaging, with no additional sterilization required for any sterile barrier system (unlike dedicated washing cycles required for reusable containers), and so the carbon footprint associated with sterilization was allocated to the instruments themselves. The disposal of packaging and reusable items (at end of life) was included, and these were assumed to enter domestic or non-infectious offensive waste streams (both processed using low-temperature incineration with energy from waste)^22^.

The purchase costs of sterile barrier systems were obtained from the SSD procurement team, and combined with the costs of decontamination to determine the financial implications of each scenario.

### Sensitivity analysis of different waste streams

The impact of sterile barrier system disposal via different waste streams was modelled, comparing high-temperature incineration (in the UK commonly used for yellow-bagged clinical waste) with recycling, to represent the highest and lowest carbon modes of healthcare waste disposal respectively^22^. Emission factors previously derived by the authors were used for high-temperature incineration^22^, and the open-loop recycled content method to account for recycling, which allocates the carbon footprint of the recycling process to the production of the recycled goods^25^.

### Analysis of streamlining instrument sets and obtaining additional instruments during surgery

The impact of removing between one and 10 instruments from a set on the carbon footprint and cost of decontamination and packaging was modelled. A standard operation was assumed to require a set containing 29 instruments (based on the RSCH retrospective audit) housed in single-use tray wrap, with loading of the decontamination machine at mean values. It was assumed that each removed instrument would be required in 20 per cent of operations (and obtained as an individually wrapped instrument), because previous studies^10^^,^^12^ of streamlining sets have suggested removal of items used less than 20 per cent of the time. Obtaining one or more instruments, either from a new set (assuming that all subsequent additional instruments could be obtained from that set, housed in single-use tray wrap) or as individually wrapped items, was also modelled, and thresholds determined at which carbon and financial costs reached parity in these scenarios.

### Optimal processes

Using the scenarios modelled, the optimum carbon footprint and cost of decontaminating and packaging an average instrument set were determined. The authors did not model how changing the proportion of lower-carbon energy would affect the cost of sterilization, but assumed that financial savings would align with carbon savings, given that UK government data estimate that generating electricity using wind and solar technologies costs half that of gas turbines^26^.

## Results

### Decontamination

Subprocesses and associated inputs for decontamination machines were summarized (*Figs S2* and *S3*). The mean duration of the washer/disinfector cycle was 45 min, and that for the sterilizer was 54 min. Total inputs for decontamination (washer/disinfector and sterilizer) per instrument set were 1.26 kWh electricity, 76 litres water, 0.3 m^3^ natural gas (3.20 kWh), and €0.04 for detergent. The carbon footprints of one typical cycle of the washer/disinfector and sterilizer were 3.74 and 12.13 kg CO_2_e per cycle respectively (*Table 1*). An audit of decontamination machines indicated that a mean average of 68 per cent of washer/disinfector and 69 per cent of sterilizer slots were occupied, and the majority of occupied machine slots (85 per cent for the washer/disinfector and 92 per cent for the sterilizer) were used for instrument sets rather than individually wrapped items (*Table S2*). Across 67 080 instrument sets processed by the SSD in the year, there was a mean of 29 items per set. Using mean machine loading and mean number of instruments per set, the carbon footprint of decontaminating surgical instruments was 52.4 g CO_2_e per instrument as part of a set (1531 g CO_2_e per set), and 145 g CO_2_e per instrument for individually wrapped items (*Table 2*).

**Table 1:** Carbon footprint of washer/disinfector and steam steriliser

	Washer/disinfector		Steam sterilizer	
	Quantity used per cycle	Carbon footprint (kg CO_2_e per cycle)	Quantity used per cycle	Carbon footprint (kg CO_2_e per cycle)
Detergent	€0.37	0.05	–	–
Electricity	8.17 kWh	2.58	4.27 kWh	1.35
Natural gas	0.36 m^3^ (13.88 MJ, 3.86 kWh)	0.83	4.35 m^3^ (167 MJ, 46.28 kWh)	9.98
Water supply and treatment	255 litres	0.27	760 litres	0.80
Total		3.74		12.13

Inputs for single cycle of each decontamination machine and their carbon footprint. CO_2_e, carbon dioxide equivalents.

**Table 2:** Carbon footprint of decontamination per instrument set and per instrument

Functional unit	Carbon footprint (g CO_2_e per functional unit)		
	Washer/disinfector	Sterilizer	Total
Instrument set	461	1070	1531
Instrument in instrument set	15.8	36.6	52.4
Individually wrapped item	59.8	85.6	145.4

CO_2_e, carbon dioxide equivalents.

**Fig. 1:**
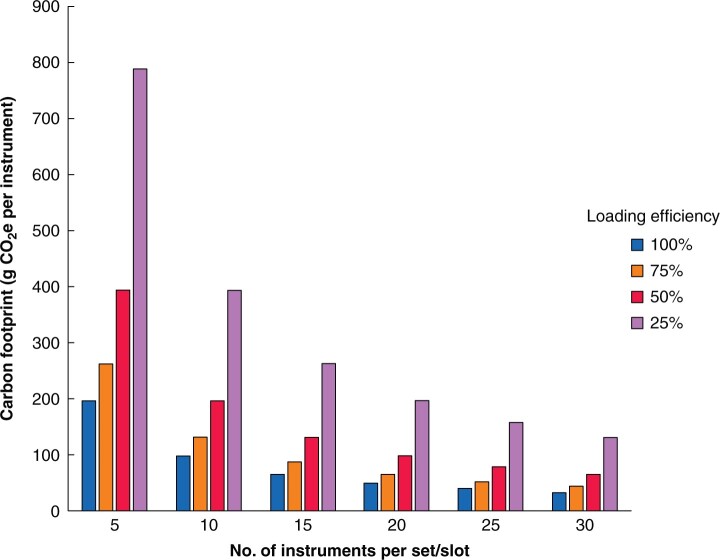
Carbon footprint of decontaminating instruments under different loading scenarios. These figures do not include the sterile barrier system. CO_2_e, carbon dioxide equivalents.

Modelling loading efficiencies indicated that, as the number of instruments per set (or per slot for individually wrapped instruments) increased, the carbon footprint decreased, and decreased further by improving the loading efficiency (proportion of slots used) (*Fig. 1*). Both the number of slots used and the number of items per slot significantly correlated with the carbon footprint (*R*^2^ = 0.678, *P* < 0.001), with both variables adding significantly to the prediction (*P* < 0.001). When the lowest observed loading was modelled (4 of 12 slots for the washer/disinfector, and 6 of 18 slots for the sterilizer, with 29 instruments per set), part-loading of machines increased the carbon footprint by a factor of 2.6 compared with typical loading (3967 *versus* 1531 g CO_2_e per set, and 137 *versus* 52 CO_2_e per instrument).

The cost of decontaminating an average set (containing 30 or fewer instruments) was €29.61 per set, and €5.60 per instrument for individually wrapped instruments.

### Sensitivity to different sources of energy for decontamination

**Fig. 2:**
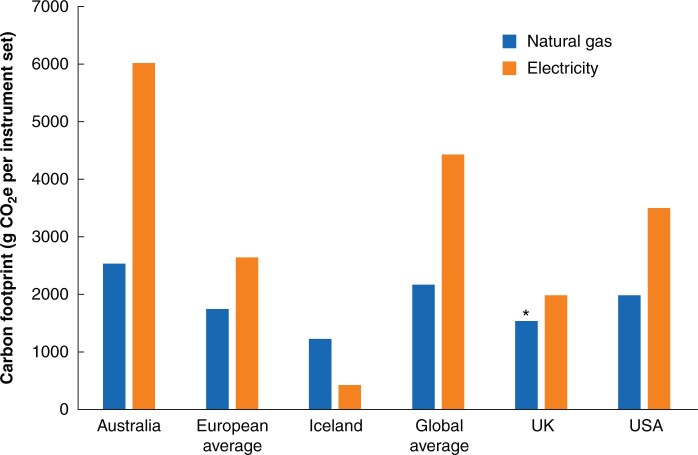
Sensitivity analysis. Total carbon footprint of decontamination (washer/disinfector plus sterilizer), modelling steam generated using either natural gas or electricity, in alternative regions. *Base scenario. CO_2_e, carbon dioxide equivalents.

The carbon footprint of decontamination (without packaging) per instrument set using natural gas to power the steam generator ranged from 1223 g CO_2_e in Iceland, to 2536 g CO_2_e in Australia, with a global average of 2166 g CO_2_e (compared with the baseline natural gas-powered UK model at 1531 g CO_2_e) (*Fig. 2* and *Table S3*). Where electricity was used to power the steam sterilizer, the carbon footprint of decontamination ranged from 422 g CO_2_e in Iceland (low-carbon energy source) to 6020 g CO_2_e in Australia (high-carbon energy source), with a global average of 4431 g CO_2_e. In most regions this was greater than the carbon footprint of decontamination with the use of natural gas to power the steam generator, owing to additional steps in generation and distribution of electricity.

In summary, integrating individually wrapped instruments into sets (instead of streamlining sets), optimising decontamination machine loading, using lower carbon energy supplies alongside recycling sterile barrier systems are strategies which can be used to reduce carbon emissions and financial costs associated with reusable surgical instruments. Surgeons and surgical teams can play a key role in driving this change through collaborating with colleagues in sterile services, to integrate supplementary items into existing sets and discourage removal of instruments from sets if these are sometimes used, and to optimise instrument set stock to be responsive to surges in demand (reducing risk of part-loading of decontamination machines). Surgeons can also champion switching to lower carbon energy sources within their own hospitals through engaging with facilities and estates departments, and seek to contract only external decontamination providers aligned with this. These activities should be alongside wider imperatives in developing sustainable surgical systems, including minimising low value care (ensuring carbon burden associated with surgery is necessary rather than avoidable), and challenging reliance on single-use instruments and linens. We also recommend that industry should investigate development of larger washers to optimise decontamination of reusable rigid containers, which would then likely become preferable to tray wrap as a sterile barrier system.

### Sterile barrier systems

The carbon footprint of the sterile barrier system per typical instrument was 25 g CO_2_e for reusable aluminium containers, 13 g CO_2_e for single-use tray wraps, and 44 g CO_2_e for flexible pouches (*Table 3*).

**Table 3:** Carbon footprint of sterile barrier system scenarios

SBS	Component	Subunit material	Weight (g)	Assumed no. of uses	Carbon footprint (g CO_2_e per use)				
					Materials	Additional decontamination*	Disposal	Total per SBS	Total per instrument†
Reusable rigid container	Basket	Stainless steel	1053.09	116	95	623	4	721	25
	Container	Aluminium	2996.54	1000					
	Identification tag	HDPE resin	8.21	46					
	Filter paper	Paper	3.55	1					
	Kit list	Paper	4.85	1					
	Tamper-proof tags	General plastic	1.78	1					
Single-use tray wrap	Basket	Stainless steel	1053.09	116	362	–	24	387	13
	Identification tag	HDPE resin	8.21	46					
	Inner wrap	Polypropylene	55.95	1					
	Kit list	Paper	4.85	1					
	Label + indicator tape	Paper	7.90	1					
	Outer wrap	Paper	64.02	1					
Flexible pouch	Identification tag	HDPE resin	8.21	46	39	–	5	44	44
	Outer pouch	Paper	4.10	1					
		General polyethylene	5.96	1					
	Inner pouch	Paper	3.68	1					
		General polyethylene	4.83	1					

*Relates to washing of reusable containers in dedicated washer**/**disinfector cycle. There was no washing involved in single-use options, and no additional impact from sterilization of any of the packaging options (allocated to instruments inside of packaging).

†Total carbon footprint of sterile barrier system (SBS) (packaging) per instrument based on mean number of instruments per set. CO_2_e, carbon dioxide equivalents; HDPE, high**-**density polyethylene.

The cost of two layers of single-use tray wrap was €1.36. The flexible pouch cost approximated €1.75 per instrument. The outlay cost of the aluminium container was €792.05, and €0.79 per use. The cost of the aluminium container achieved parity with tray wrap after 591 uses, and with flexible pouches after 454 uses.

### Sensitivity of sterile barrier systems to different sources of waste disposal

Compared with baseline (use of low-temperature incineration with energy from waste), disposal by high-temperature incineration increased the carbon footprint of reusable rigid containers across their life cycle by only 3 per cent (to 25.4 g CO_2_e per instrument), and recycling reduced the carbon footprint by only 0.5 per cent (to 24.5 g CO_2_e per instrument). Larger differences were found for single-use sterile barrier systems (*Table S4*); there was a 33 per cent increase in the total carbon footprint with high-temperature incineration for single-use tray wrap (to 18 g CO_2_e per instrument) and a 55 per cent increase for flexible pouches (to 68 g CO_2_e per instrument). There was a 6 per cent decrease in carbon footprint with use of recycling for tray wrap (to 12 g CO_2_e per instrument) and a 10 per cent decrease for flexible pouches (to 39 g CO_2_e per instrument).

### Total carbon footprint and cost of decontamination and packaging

**Fig. 3:**
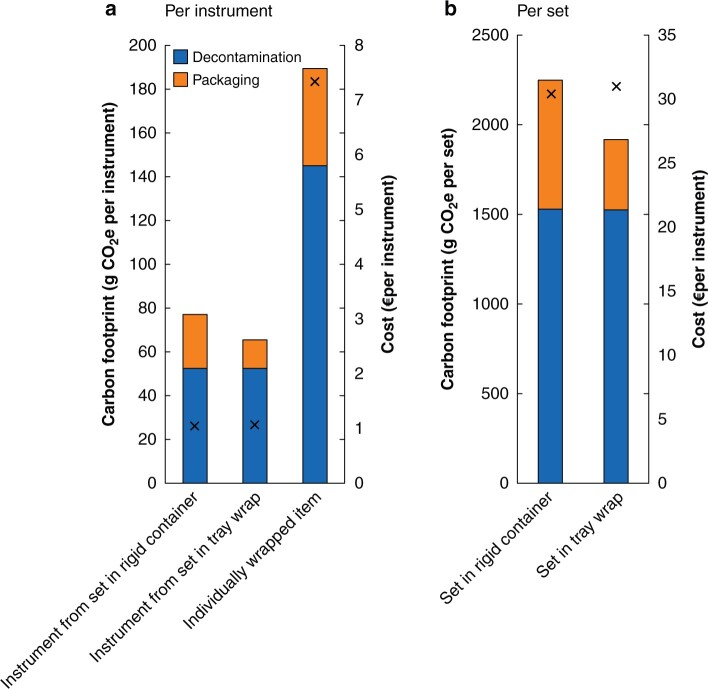
Total carbon footprint and cost of decontaminating and packaging reusable instruments per instrument and per set. Bars show carbon footprint and crosses indicate financial cost **a** per instrument and **b** per set. Each bar is split in two to demonstrate the contribution of the decontamination process and packaging to the carbon footprint. CO_2_e, carbon dioxide equivalents.

The total carbon footprint and cost of decontaminating and packaging instruments (baseline model and assumptions), was 77 g CO_2_e (€1.05) per instrument housed in aluminium containers (2252 g CO_2_e, €30.41 for the whole set), 66 g CO_2_e (€1.07) per instrument in tray wrap (1918 g CO_2_e, €30.98 for the whole set), and 189 g CO_2_e (€7.35) per individually wrapped instrument (*Fig. 3*). In summary, both the carbon footprint and the financial cost of an individually wrapped instrument was greater than that for an instrument in a set packaged in a rigid container, which in turn had a greater carbon footprint per use than an instrument in a set packaged in tray wrap (and substantially the same financial cost).

### Streamlining instrument sets and obtaining extra instruments during surgery

**Fig. 4:**
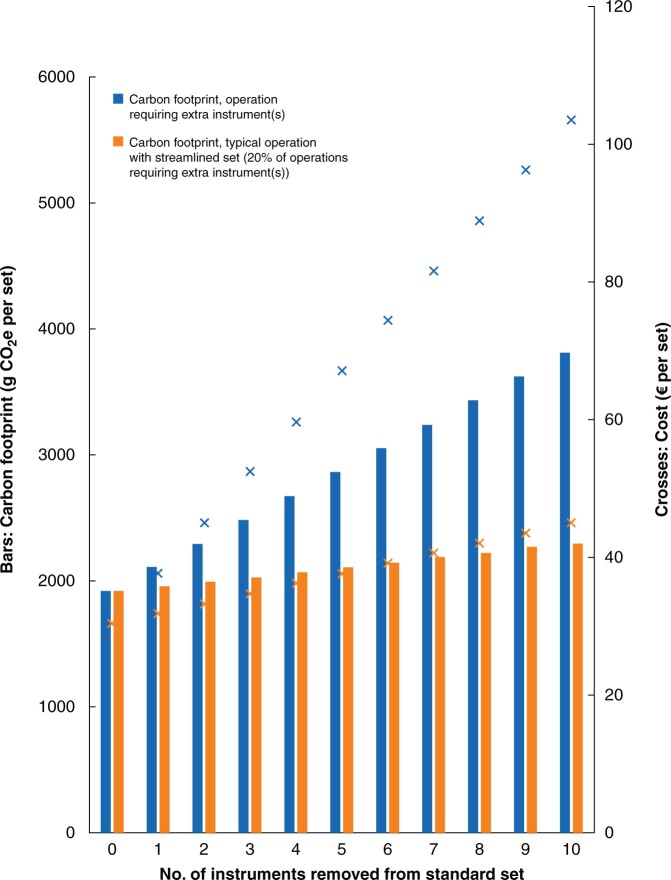
Impact of streamlining instrument sets on the carbon footprint and cost of decontamination and packaging of reusable instruments. Based on a standard set containing 29 instruments, housed in single-use tray wrap, with loading of decontamination machine at mean values. Bar graph shows carbon footprint and crosses indicate financial cost. CO_2_e, carbon dioxide equivalents.

Removing items from a set proportionately increased the carbon footprint and cost of decontamination and packaging reusable instruments (*Fig. 4*). For operations requiring the removed instrument(s) as individually wrapped items (20 per cent of procedures), this generated an additional 189 g CO_2_e and cost an extra €7.34 per item. The carbon footprint increased by a mean of 38 g CO_2_e (costing an additional €1.47) per item removed across all operations requiring the streamlined set.

When obtaining extra instruments during surgery, the carbon footprint of decontamination and packaging was lower when additional instruments were obtained as individually wrapped items when 10 or fewer items were required, and the costs lower when four or fewer items were required. Above these thresholds, carbon and financial costs were lower when instruments were obtained by opening an additional set.

### Optimal processes

**Fig. 5:**
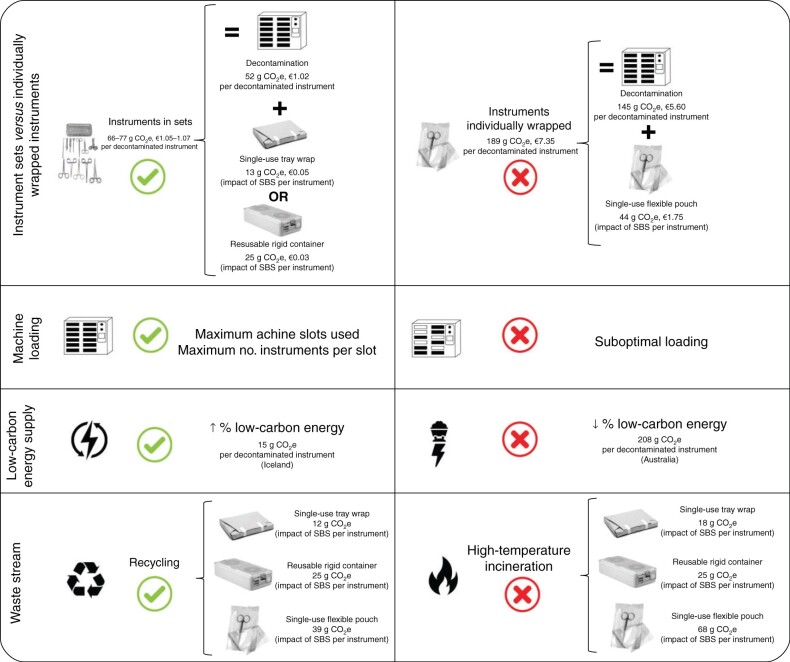
Optimizing carbon footprint and financial cost of decontaminating and packaging reusable instruments. Low-carbon energy supply models assume electricity-powered steam generation. CO_2_e, carbon dioxide equivalents; SBS, sterile barrier system.

The carbon footprint and cost of instrument decontamination and packaging were optimized through four strategies (*Fig. 5*): processing instruments in sets rather than individually wrapped; maximal loading of the decontamination machine (100 per cent slots used, 30 instruments per slot); increasing the proportion of low-carbon energy supply; and recycling of the sterile barrier system.

The choice of a reusable rigid container for housing the set was associated with lowest financial cost (marginally lower than that with use of single-use tray wrap), whereas employing single-use tray wrap resulted in the lowest carbon footprint. Optimum financial costs (assuming use of reusable rigid containers) equated to €30.41 per set, and €1.01 per instrument, and the optimum carbon footprint (assuming use of single-use tray wrap in the UK) was 1348 g CO_2_e per set, and 45 g CO_2_e per instrument. Where Icelandic electricity was modelled, the carbon footprint was reduced further to 633 or 1141 g CO_2_e per set (assuming electricity- or natural gas-fuelled steam generation respectively), equating to 21 or 38 g CO_2_e per instrument.

## Discussion

Typically, the principal determinant of the life-cycle carbon footprint of reusable surgical instruments is the decontamination process, which is responsible for up to 85 per cent of the carbon footprint of reusable surgical scissors^27^, and almost all GHG emissions associated with reusable laryngoscope blades and handles^15^. The carbon footprint of processes upstream and downstream of decontamination (product manufacture, distribution, and waste) apportioned per instrument use can be reduced by increasing the number of uses, but the decontamination process will remain a constant hotspot. To minimize the environmental impact of reusable instruments, it is important therefore that the surgical team understands how to mitigate this impact.

This study indicated that the carbon footprint of decontaminating and packaging an average reusable instrument in the UK can be reduced to 45 g CO_2_e, achieved by processing instruments as part of sets (housed in single-use tray wraps), and maximizing the loading of decontamination machines. This optimal process represents a 31–42 per cent reduction compared with average loading of instrument sets (packaged in single-use tray wrap and reusable aluminium containers respectively), and a 76 per cent reduction compared with individually wrapped instruments. The absolute carbon footprint of decontamination is also dependent on the energy source used, and so could be further optimized by use of lower-carbon energy sources, although this does not affect the components of the optimal process outlined above.

The preparation of instruments, either as individually wrapped items (189 g CO_2_e per instrument) or in sets (66–77 g CO_2_e per instrument), had a two- to three-fold impact on the carbon footprint. Instruments are typically individually wrapped because of infrequent use, convenience, or habit. Several authors^28^^,^^29^ have suggested that the environmental impact of an operation can be reduced by streamlining instrument sets, but the results of this study do not support such assertions. Here, streamlining sets was found counterintuitively to increase the carbon footprint and financial costs. When decontamination of instruments is undertaken by loading of sets into a machine, the carbon footprint of that decontamination cycle is almost unaffected by the number of instruments in that set, but processing instruments separately significantly increased the per-instrument cost and carbon footprint. Surgeons should therefore request that instruments even occasionally used in an operation should remain in sets (although instruments never or very rarely used can be removed), and that individually wrapped instruments are integrated into appropriate sets where possible. Where integration into existing sets is not possible (for example, because of the size or number of additional instruments) and more than eight instruments are frequently required, these instruments should be collated to form a new set. This finding does not preclude the notion that some individually wrapped instruments should remain available in case they are needed owing to intraoperative instrument damage or loss of sterility, because the associated carbon footprint remains preferable to that of opening a whole new set when 10 or fewer instruments are required, and four or fewer from a financial perspective. These data apply to reusable instruments; in contrast, for single-use pre-prepared sets, streamlining may confer benefits, for example reducing the carbon footprint of a hysterectomy by up to 46 per cent^30^. However, it should be recognized that, in almost all circumstances, single-use instruments carry greater carbon and financial costs than reusable equivalents^15^^,^^27^.

The carbon footprint of decontaminating and packaging reusable instruments was sensitive to machine loading. Although it is inevitable that machine slots may sometimes be unfilled to meet operational demands, this should be minimized. These findings are consistent with those of another study^17^ which found that heavier machine loads were more efficient, although the number of instruments housed per slot would be imperfectly correlated with the weight of those items. Surgeons can work with colleagues in SSDs and the wider surgical team to optimize instrument flow and stock levels, minimizing the need for last-minute decontamination using partially filled machines.

Although the cost charged to surgical departments for sterilization of instrument sets was the same regardless of the packaging used, this study showed that the per-use purchase and disposal cost of using a reusable rigid container was around half that of single-use tray wrap per use. Depending on local arrangements (and use of on-site *versus* off-site decontamination services) this may confer a financial saving; for example, one hospital in the USA estimated savings of US $51000 (€42 072; exchange rate 16 June 2021) per year by switching from tray wrap to reusable containers^31^. Switching to reusable packaging may also help meet obligations to reduce plastic use in healthcare, as the outer layer of tray wrap is typically made from plastics such as polypropylene, and so switching to reusable metal containers would also reduce waste disposal costs (120 g less waste per set). However, the carbon footprint of using single-use tray wraps (13 g CO_2_e per instrument, 387 g CO_2_e per set) was around half that of using reusable aluminium containers as the sterile barrier system (25 g CO_2_e per instrument, 721 g CO_2_e per set), and a three-fold reduction compared with wrapping instruments individually in pouches (44 g CO_2_e per instrument). The carbon footprint of employing reusable containers was principally determined by the washing process (86 per cent of the impact of the reusable container itself), and this may be reduced by use of larger washer/disinfector machines manufactured specifically for the washing of reusable rigid containers, which are likely to be more efficient, and by ensuring that the tray is of the smallest sufficient size. If this were enabled, it is likely that the carbon and financial costs of reusable containers would become less than that of single-use tray wraps, and so is something the industry should be looking to develop.

Individually wrapped instruments are often double-wrapped in two flexible pouches, in part for the convenience of theatre staff, yet the Association of Surgical Technologists^32^ recommends this practice only when packaging multiple items or multicomponent items. Outer flexible pouches for individually wrapped instruments are typically one size larger than the inner pouch, so switching to single pouches more than halves the associated carbon footprint, financial cost, and waste. A comparison of the clinical performance of different sterile barrier systems is beyond the scope of the present discussion, but if (as would be expected) these comply with sterility assurance standards^8^, housing instruments in sets rather than individually in flexible pouches should be standard hospital policy wherever feasible.

The carbon footprint of decontamination varied with differences in carbon intensity of energy and natural gas supply, with a 14-fold difference between high- and low-carbon energy sources when using electricity-fuelled generation of steam. The source of energy does not affect the optimal process for decontamination and sterilization of instruments, but the finding does illustrate that optimizing processes to reduce the carbon burden is still at the mercy of national energy strategy, and that increasing the availability of lower-carbon electricity must be an allied strategy in attempts to reduce the carbon footprint. The recommendations of this study are broadly generalizable, although country- or region-specific figures should be applied by those wishing to evaluate data specific to their context.

Recycling was associated with a 6–10 per cent reduction in the carbon footprint of single-use sterile barrier systems (tray wrap and flexible pouches respectively). This open-loop recycling model^25^ assumed that waste materials were downcycled and used for the generation of other items, for example production of toolboxes, bottles, and bins from polypropylene tray wrap^33^. The manufacture of sterile barrier systems from recycled materials in a closed-loop model^25^ would further reduce the carbon footprint (owing to the reduced acquisition of virgin raw materials), and innovation towards this should be encouraged. Conversely, use of high-temperature incineration (common for clinical waste) increased the carbon footprint of tray wraps by one-third, and of flexible pouches by over half, highlighting the opportunity to explore optimal waste disposal^22^. Nevertheless, the authors also point to their earlier recommendation that larger washing machines for reusable containers may prove a better strategy for reducing the carbon and financial cost of sterile barrier systems, and also eliminate much of this associated waste.

Optimizing the decontamination and packaging of surgical instruments at scale could help us meet net zero carbon within surgery and save money. For example, if one additional individually wrapped instrument were required 20 per cent of the time across the 313 million annual surgical procedures performed globally^1^, based on the authors’ UK data, this would generate an additional 11 850 tonnes of CO_2_e (equivalent to a single passenger flying from London to New York return 4900 times).

The present findings relating to the optimal process are also applicable to decontamination of instruments used outside of the operating theatre, including, for example, those employed in outpatient settings, or laryngoscope blades used in anaesthesia. These principles can also be applied to mitigating the carbon footprint of reprocessing reusable linens, such as surgical gowns and drapes, by optimizing machine loading and using low-carbon energy.

This study has a number of limitations. Cost calculations were based on amounts charged to surgical departments, with no direct measure of staff time in the sterilization department or operating theatre. Estimates of carbon footprints are dependent on the boundaries set and assumptions made. The decontamination machines modelled were around 10 years old, and newer versions may be more efficient or have different loading capacities. The frequency and annual volume of decontamination may differ at other hospital sites, as well as the composition of instrument sets, or of specific sterile barrier systems. Some hospitals use centralized off-site services, rather than the on-site services modelled here. However, when additional transportation for off-site sterilization was modelled, based on a 160-km round trip by road, this increased the carbon footprint by only 1–6 per cent (*Table S5*). Such differences in process would change values for the data presented in this paper, but it seems unlikely that they would alter the overall optimal strategy for decontamination outlined here.

This study focused on steam sterilization, which is a standard method in the UK^7^. Other methods of microbial inactivation have different environmental and financial costs. For example, a study^14^ in Australia found that ethylene oxide sterilization of a single-use ureteroscope during manufacture had a carbon footprint of just 7 per cent of that for steam sterilization of a reusable equivalent, and a study^5^ in India estimated that the electricity consumption of rapid high-pressure steam sterilization (also known as flash autoclaving, or benchtop sterilization) was around one-quarter of that of standard steam sterilization. Such methods are not widely used in the UK; flash autoclaving is less reliable than standard steam sterilization^34^, and ethylene oxide sterilization is usually used only for sensitive materials, in part because this compound poses risks to human and environmental health^35^.

## Supplementary Material

znab406_Supplementary_Data

## Notes

Presented to the Proceedings from Life Cycle Innovation Conference, Berlin, Germany, August 2020
