# Supplementary material for: Minimising carbon and financial costs of steam sterilisation and packaging of reusable surgical instruments
Source: Br J Surg. 2021 Nov 28;109(2):200–10. doi: 10.1093/bjs/znab406 (PMC10364739; doi:10.1093/bjs/znab406)
Supplement: znab406_Supplementary_Data [file znab406_supplementary_data.docx]

Table S1: Emission factors

| Process/ product | | Emission factor | | Emission factor unit | Source |
| --- | --- | --- | --- | --- | --- |
|  |  | Component | Total |  |  |
| Aluminium | | 6.72 | 6.72 | kg CO_2_e/ kg | ICE v3^1^ |
| Domestic/ non-infectious offensive hospital waste (low temperature incineration with energy from waste | | **171.78** | **171.78** | kg CO_2_e/ tonne | Rizan et al^2^ |
| Clinical waste | | **1074.13** | **1074.13** |  |  |
| General polyethylene | | 2.54 | 2.54 | kg CO_2_e/ kg | ICE v3^1^ |
| General plastic | | 3.31 | 3.31 | kg CO_2_e/ kg | ICE v3^1^ |
| High density polyethylene (HDPE) resin | | 1.93 | 1.93 | kg CO_2_e/ kg | ICE v3^1^ |
| Manufacture of soap and detergent | | 0.17 | 0.17 | £ | Small World Consulting^3^ |
| UK natural gas | Combustion | 2.03 | 2.29 | kg CO_2_e/ m^3^ | DEFRA/BEIS^4^ |
|  | Well to tank | 0.26 |  |  |  |
| Alternative natural gas well to tank (combustion as above) | European | 0.40 |  | kg CO_2_e/ m^3^ | SimaPro^5^ |
|  | Global average | 0.42 |  |  |  |
|  | US | 0.51 |  |  |  |
| Paper | | 1.49 | 1.49 | kg CO_2_e/ kg | ICE v3^1^ |
| Polypropylene oriented film* | | 3.43 | 3.43 | kg CO_2_e/ kg | ICE v3^1^ |
| Stainless steel | | 6.145 | 6.145 | kg CO_2_e/ kg | Small World Consulting^3^ |
| UK electricity | Generation | 0.26 | 0.32 | kg CO_2_e/ kWh | DEFRA/BEIS^4^ |
|  | Transmission and distribution | 0.02 |  |  |  |
|  | Generation well to tank | 0.04 |  |  |  |
|  | Transmission and distribution well to tank | 0.003 |  |  |  |
| Alternative electricity | Australian | 0.99 | | kg CO_2_e/ kWh | SimaPro^5^ |
|  | European | 0.42 | |  |  |
|  | Global average | 0.73 | |  |  |
|  | Icelandic | 0.06 | |  |  |
|  | US | 0.57 | |  |  |
| Water | Supply | 0.34 | 1.05 | kg CO_2_e/ m^3^ | DEFRA/BEIS^4^ |
|  | Treatment | 0.71 |  |  |  |
| Transportation | Heavy goods vehicle (diesel, average laden) | 0.11 | 0.14 | kg CO_2_e/ tonne.km | DEFRA/BEIS^4^ |
|  | Well to tank | 0.02 |  |  |  |

Emission factors used to determine the carbon footprint of decontamination and packaging of reusable surgical instruments. *Note polypropylene oriented film chosen as closest fit within ICE database for non-woven polypropylene (SimaPro^5^ estimate for non-woven polypropylene=3 kg CO_2_e/ kg)

References:

1. Inventory of Carbon and Energy v3.0 [database]. Jones C, Hammond G: University of Bath; 2019.

2. Rizan C, Bhutta M, Reed M, et al. The carbon footprint of waste streams in a UK hospital.  Journal of Cleaner Production. 2020;286:125446

3. Carbon Factors Dataset version 1.5 [database]. Small World Consulting: Lancaster University; 2018.

4. UK Government GHG Conversion Factors for Company Reporting [database]. Department for Environment, Food and Rural Affairs/ Department for Business, Energy & Industrial Strategy; 2019.

5. SimaPro Version 9.10, Ecoinvent (version 3.6) [database]. PRé Sustainability, Amersfort, Netherlands, 2019.

Table S2: Decontamination machine loading audit results

| ****Unit**** | ****Metric (all per cycle)**** | ****Washer/ disinfector**** | ****Steriliser**** |
| --- | --- | --- | --- |
| **Total number of instruments** | **Mean**  **(95% CI)** | **159.00**  **(120.07-197.93)** | **309.90**  **(192.90-426.90)** |
| **Total slots used** | **Mean**  **(95% CI)** | **8.10**  **(6.21-9.98)** | **11.33**  **(8.81-13.87)** |
|  | **Mean proportion of slots available used (%)** | **67.50** | **68.54** |
| **Slots containing individually wrapped items** | **Mean**  **(95% CI)** | **1.20**  **(0.24-2.16)** | **1.00**  **(0.23-1.77)** |
|  | **Mean proportion of used slots (%)** | **14.81** | **8.11** |
| **Number of instruments per slot used for individually wrapped items** | **Mean**  **(95% CI)** | **7.08**  **(3.75-10.42)** | **12.5**  **(7.06-17.94)** |
| **Slots containing instrument sets** | **Mean**  **(95% CI)** | **6.9**  **(5.68-8.12)** | **11.34**  **(8.81-13.87)** |
|  | **Mean proportion of used slots (%)** | **85.19** | **91.89** |
| **Number of instruments per slot used for instrument sets** | **Mean**  **(95% CI)** | **21.81**  **(18.98-24.64)** | **23.88**  **(18.57-29.20)** |

95% CI (confidence intervals) in parenthesis

**Table S3: Sensitivity analysis**

**Legend:** Total carbon footprint of decontamination (washer/disinfector plus steriliser), modelling steam generated using either natural gas or electricity, and alternative regions, CO_2_e= carbon dioxide equivalents, *=base scenario

| **Region** | **Steam generation energy source** | **Carbon footprint (g CO_2_e/ functional unit)** | | |
| --- | --- | --- | --- | --- |
|  |  | **Instrument set** | **Instrument in instrument set** | **Individually wrapped item** |
| Australia | Natural gas | 2,536 | 86.8 | 260 |
|  | Electricity | 6,020 | 206 | 556.8 |
| European average | Natural gas | 1,739 | 59.5 | 167.8 |
|  | Electricity | 2,639 | 90.3 | 244.4 |
| Iceland | Natural gas | 1,222 | 41.8 | 107.8 |
|  | Electricity | 421.3 | 14.4 | 39.5 |
| Global average | Natural gas | 2,166 | 74.1 | 217 |
|  | Electricity | 4,431 | 151.6 | 410 |
| UK | Natural gas* | 1,531 | 52.4 | 145.4 |
|  | Electricity | 1,989 | 68 | 184.2 |
| US | Natural gas | 1,990 | 68.1 | 195.4 |
|  | Electricity | 3,499 | 119.7 | 323.9 |

**Table S4: Sensitivity analysis of total carbon footprint of sterile barrier systems to differing waste streams**

**Legend:** Total results presented here relate to materials, additional decontamination plus waste disposal of sterile barrier systems (packaging), under two alternative waste disposal scenarios. CO_2_e= carbon dioxide equivalent

| **SBS** | **Carbon footprint (g CO_2_e/ use)** | | | |
| --- | --- | --- | --- | --- |
|  | **Total per SBS** | | **Total per instrument** | |
|  | **High temperature incineration** | **Recycling** | **High temperature incineration** | **Recycling** |
| Reusable rigid container | 741 | 717 | 25 | 25 |
| Single-use tray wrap | 515 | 362 | 18 | 12 |
| Flexible pouch | N/A | N/A | 68 | 39 |

**Table S5: Carbon footprint of transport to offsite decontamination site**

**Legend:** CO_2_e= carbon dioxide equivalents

Assumptions:

The mean weight of instruments across three typical sets used for common operations (tonsillectomy set, minor op set used for carpal tunnel decompression, and basic major orthopaedic set) was 66.7 g. The mean number of instruments per set across sets was 29 (determined using a retrospective audit of instrument decontaminations over one year at our local hospital conducted 1/7/18- 30/6/19). A typical instrument set was therefore assumed to contain 2 kg instruments, and a single instrument was assumed to weigh 66.7 g.

We assumed that the offsite decontamination centre was located 80 km away from the hospital, and that transportation involved 160km round trip in an average diesel heavy goods vehicle (average laden).

| **Functional unit** | **Weight (g)** including sterile barrier system and instrument(s) within | **Carbon footprint of transportation (g CO_2_e)** |
| --- | --- | --- |
| Instrument set housed in reusable rigid container | 6,068 | 132.69 |
| Instrument set housed in single-use tray wrap | 3,194 | 69.84 |
| Individually wrapped instrument housed in flexible pouch | 93 | 2.04 |

Figure S1: System boundary

System boundary for carbon footprint of decontamination and packaging reusable surgical instruments. Processes included were energy and materials required by the washer/ disinfector, steriliser, and sterile barrier system, alongside disposal of materials used within the sterile barrier system. Energy and materials for sterile barrier system included any additional decontamination resulting from choice of packaging, which included washing of rigid containers in dedicated cycles in the washer/disinfector, but there was no additional impact from sterilisation of the sterile barrier system. Capital goods, hospital infrastructure, and production and disposal of the surgical instruments were excluded.

Figure S2: Processes involved in washer/ disinfector cycle and input requirements

**The washer/ disinfector machine (far right) is reliant on a number of other machines (grey boxes). The inputs (energy, fuels, water and chemicals) for each of these machines is illustrated in white boxes, with the input requirements of each machine per cycle shown in red.**

Figure S3: Processes involved in steriliser cycle and input requirements

**The steriliser machine (far right) is reliant on a number of other machines (grey boxes). The inputs (energy, fuels, water and chemicals) for each of these machines is illustrated in white boxes, with the input requirements of each machine per cycle shown in red.**
